# Impact of preoperative SYNTAX Scores on short-term outcome following coronary artery bypass grafting surgery in the patients with multi-vessels coronary artery disease

**DOI:** 10.1186/s43044-020-00071-3

**Published:** 2020-07-02

**Authors:** Bassem Adel Ramadan, Mohamed Ahmed Zaki, Wahid Gamal El Din Etman, Mohamed Mostafa Agha, Mohamed Ahmed Sobhy, Wael Mahmoud Hassanein

**Affiliations:** Faculty of Medicine, Alexandria University, Sultan Hussein Street, Al Khartoum Square, Alexandria, Egypt

**Keywords:** CABG, In-hospital mortality, RRT, SYNTAX Score I, SYNATAX Score II, MI, MACCE, Short-term outcome

## Abstract

**Background:**

SYNTAX Scores I (SSI) assesses the complexity of CAD; SYNTAX Score II (SSII) uses both SSI and other clinical variables, in estimation of 4 years mortality following both coronary artery bypass grafting surgery (CABG) and percutaneous coronary intervention (PCI) and gives recommendations for the best revascularization strategy in a specific patient. Our aim is to investigate the impact of both SYNTAX Scores on short-term outcome following CABG.

**Results:**

Prospectively, we studied 150 patients with multi-vessels coronary artery disease, referred to perform, elective primary isolated CABG. All cases performed on pump CABG with aortic cross clamping, then followed up for 90 days postoperatively, for onset of mortality from all causes, myocardial infarction (MI), stroke, mediastinitis, and need for renal replacement therapy (RRT).

SSI showed a statistically significant association with in-hospital and 90 days mortality, MI, and mediastinitis (*P* = < 0.001, 0.015, 0.045 respectively); SSII showed a statistically significant association with in-hospital mortality and 90 days mortality and need for renal replacement therapy (*P* = 0.007, 0.043, 0.012 respectively); SSI is independent risk factor for overall mortality (OR 1.192, 95% CI 1.018–1.396) (*P* = 0.029) and MI (OR 1.182, 95% CI 1.016–1.375).

**Conclusions:**

SYNTAX Scores are good predictors of short-term outcome after CABG; increased SSI was associated with increased mortalities (in-hospital and total 90 days), MI and mediastinitis, increased SSII associated with increased mortalities (in-hospital and total 90 days), and need for RRT; SSI is independent risk factor for mortality and MI.

## Background

Coronary artery disease (CAD) is the most common cause of death in the developed countries and is representing the commonest pathological entity facing the adult cardiologists and cardiac surgeons, with treatment options including optimized medical treatment, percutaneous Coronary Intervention (PCI) and/or coronary artery bypass grafting surgery (CABG) [1, 2].

Choice of the best revascularization strategy should be carried out by a multidisciplinary heart team, to ensure choice of the most suitable and durable revascularization method with focusing on the concept of complete revascularization, which leads to marked improvement of symptoms and reduction of the procedure-associated mortality and morbidity [3].

Marked improvement of PCI technologies in the past years with continuous emergence of newer generations of drug eluting stents resulted in replacement of CABG by PCI in many situations especially in the settings of acute coronary syndromes. CABG is still the revascularization strategy of choice in the management of patients with left main (LM) disease, diabetic patients with multi-vessels disease (MVD), and patients with complex anatomy of coronary arteries. As in these patients, CABG is associated with better long-term outcome, in the form of lower incidence of mortality and major adverse cardiac events [4–6].

Preoperative risk assessment is an integral part of the management of patients prepared for CABG, as the estimated surgical risk may affect the choice of the revascularization strategy. There are many valid risk scores that used for this purpose; both EuroSCORE II and STS scores are effective and accurate in estimation of in-hospital and 30 days mortality post cardiac surgery but sharing a common disadvantage of neglecting the anatomical complexity of the coronary arteries [7–9].

SYNTAX Score I (SSI) is an anatomical scoring system that was derived from the SYNTAX trial (Synergy between PCI with Taxus and Cardiac Surgery), used for assessment of the anatomical complexity of coronary arteries, on the basis of SSI the complexity classified into 3 categories: low SSI (0–22), intermediate (23–32), and high (more than 33). In the patients with low SSI, PCI is not inferior to CABG (better option) as an option for revascularization, while in the patients with high SSI, CABG is the better option, as PCI in these patients associated with higher incidence of long-term mortality and MACCE. Main disadvantage of SSI is its neglection to the clinical conditions and comorbidities of the patient that may affect the outcome of surgery [4, 10, 11].

SYNTAX Score II (SSII) combines both SSI and 7 clinical variables (age, sex, ejection fraction (EF), chronic obstructive pulmonary disease (COPD), peripheral vascular disease (PVD), unprotected LM disease, and creatinine clearance), in estimation of 4 years mortality following both CABG and PCI in a specific patient and gives a recommendation for revascularization: CABG, PCI, or both [6, 9, 12].

The relationship between SSI and SSII with the outcome of CABG and/or PCI was proved to be mainly on the long-term follow-up, but the effect of these scores on the short-term outcome is still not clear, especially following CABG, which is our aim in this study.

## Methods

The aim of our study was to assess the impact of preoperative SYNTAX Scores on short-term outcome following CABG in the patients with MVD. This research was approved by our institution Ethics Committee, and written informed consent was obtained from all the participants included in this study.

We conducted a prospective, observational study on 150 patients with MVD, referred to our institute to perform, elective isolated CABG, in the period extended from August 2018 to November 2019. Patients with previous cardiac surgery, on maintenance hemodialysis, and those were not compliant during the follow-up period were excluded from the study. Preoperative data reviewed and both SYNTAX Scores were calculated using on-line program.

SSI calculated as a sum of points assigned to each individual lesion identified in the coronary arteries, in the coronary angiography with > 50% diameter narrowing in the vessels more than 1.5 mm diameter. The specific weight for each lesion differs according to the affected vessel and the dominance of coronary circulation.

Additional points added to total occlusion more than 3 months, calcified vessels, severely tortious vessels, bifurcation and trifurcation lesions, bridging collaterals, intraluminal thrombus, long segment lesion, aorto-ostial lesions, and diffusely diseased vessels. After introduction of the data, the program gives the SSI value.

SSII calculated by the same program, by combining the SSI with other clinical variables (age, sex, COPD, PVD, creatinine clearance, LV ejection fraction, and LM disease).

SSII calculator gives SSII for both CABG and PCI, expected 4 years mortality following both CABG and PCI and gives a recommendation for the revascularization strategy, whether CABG, PCI, or both.

Our study population was subjected to on pump CABG, with aortic cross clamping and cold crystalloid cardioplegia; we used skeletonized left internal mammary artery (LIMA) to left anterior descending coronary (LAD) anastomosis in all cases, saphenous vein graft used for the other anastomotic sites, and skeletonized right internal mammary artery (RIMA) used as the second arterial graft, in some selected cases.

Intraoperative transesophageal echocardiography (TEE) is used for assessment of myocardial function and detection of new regional wall motion abnormalities (RWMA); it was done after induction of anesthesia and following weaning from cardiopulmonary bypass.

We followed up our patients for 90 days postoperatively for onset of all causes mortality, myocardial infarction (MI), stroke, mediastinitis, and need for renal replacement therapy (RRT).

In diagnosis of perioperative MI, we depended on a combination between elevated CKMB/total CK ratio more than 10%, together with new ECG changes (ST segment elevation, new left bundle block, Q wave, ventricular arrhythmias), clinical situation of the patient (hemodynamic instability, increased need for inotropes, cardiac arrest), and/or new RWMA that detected either by TEE or TTE, either intraoperatively or in ICU.

In patients who experienced significant ECG changes and/or hemodynamic instability in the perioperative period, the ratio between CKMB and total CK investigated, and immediate echo was done (TTE or TEE) according to the situation to detect new RWMA. A combination between the previously mentioned findings was diagnostic for perioperative MI.

Coronary angiography done for the cases presented with late onset MI after hospital discharge. RRT was initiated early in anuric patients who did not respond to maximum diuresis, oliguric patients with doubling of their serum creatinine within 24 h, and oliguric patients with hyperkalemia who did not respond to anti-hyperkalemic measures. Newly developed neurological deficit with positive findings on brain imaging were diagnostic for stroke.

The data collected during the follow-up period were tabulated and prepared for the statistical analysis; these data did not include any private or personal information of our patients.

### Statistical analysis of the data

Data were fed to the computer and analyzed using the IBM SPSS software package version 20.0*.* (Armonk, NY: IBM Corp). Qualitative data were described using number and percent. The Kolmogorov-Smirnov test was used to verify the normality of distribution. Quantitative data were described using range (minimum and maximum), mean, standard deviation, median, and interquartile range (IQR). Significance of the obtained results was judged at the 5% level. The used tests were Chi-square test, Fisher’s Exact, Student *t* test, *F* test (ANOVA) (analysis of variance), Pearson coefficient, and Mann Whitney test

## Results

We studied a total number of 150 patients with MVCAD; the preoperative data of our population are summarized and distributed in Table 1.

**Table 1:** Distribution of the studied cases according to the preoperative data (*n* = 150)

	No.	%
**Sex**		
Female	53	35.3
**Age (years)**		
Mean ± SD	60.10 ± 9.21	
**DM**	120	80.0
**LM disease**	48	32.0
**PVD**	42	28.0
**COPD**	58	38.7
**EuroSCORE II**		
Mean ± SD	1.49 ± 0.88	
**EF %**, mean ± SD	55.50 ± 8.27	
**Cr. clearance** (mean ± SD)	93.22 ± 20.88	
**SSI** (mean ± SD)	26.24 ± 5.84	
**SSII CABG** (mean ± SD)	26.96 ± 10.85	

*SSI* SYNTAX Score I, *cr. clearance* creatinine clearance, *SSII* SYNTAX Score II, *LM* left main, *COPD* chronic obstructive pulmonary disease, *MI* myocardial infarction, *PVD* peripheral vascular disease

SYNTAX Score II recommended both CABG and PCI to 90 patients of our cohort (60%), CABG only for 46 (30.7%), while PCI only recommended for 14 patients (9.3%). By following up these PCI recommended patients, we found among them one case had in-hospital mortality from MI. All our patients performed CABG, with mean graft number 2.95 ± 0.70, and we found a statistically significant association between the increasing SSI and number of bypass grafts (P = 0.005).

Table 2 summarizes the incidence of mortalities and the studied complication, in our study population. As regards MI, 7 of them were in the perioperative period (4.6% perioperative MI), while the remaining 3 cases presented with late onset MI, after hospital discharge, also, one case with stroke presented in day 50 postoperatively and the remaining 4 cases in the perioperative period.

**Table 2:** Incidence of mortalities and complications in study population (*n* = 150)

	No.	%
**Overall mortality**	10	6.7
**In-hospital mortality**	8	5.3
**MI**	10	6.7
**Stroke**	5	3.3
**Need for RRT**	3	2.0
**Mediastinitis**	5	3.3

The distribution of mortalities in our study was as follows: 5 cases died from perioperative MI, 2 cases from respiratory failure, and one case from stroke (hemorrhagic transformation with midline brain shift). The additional 2 cases died after hospital discharge during follow-up period; one case from late onset MI and the other one were with undetermined etiology.

Table 3 summarizes the relation between preoperative SSI with the incidence of mortalities and studied complications; from this table, we had a statistically significant association between increasing SSI and the incidence of in-hospital mortality, total 90 days mortality, MI, and Mediastinitis.

**Table 3:** Relation between preoperative SSI with the mortalities and studied complications (*n* = 150)

	***N***	%	SSI (mean ± SD)		***P***
			Complicated	Non complicated	
**In-hospital mortality**	8	5.3	31.63 ± 1.87	25.94 ± 5.85	< 0.001^*^
**90 days mortality**	10	6.7	30.75 ± 2.91	25.92 ± 5.87	< 0.001^*^
**MI**	10	6.7	30.55 ± 4.79	25.93 ± 5.80	0.015^*^
**Stroke**	5	3.3	31.0 ± 4.18	26.08 ± 5.83	0.064
**Need for RRT**	3	2	29.0 ± 10.04	26.18 ± 5.77	0.410
**Mediastinitis**	5	3.3	27.70 ± 1.10	26.19 ± 5.93	0.045^*^

*P P* value for association between different categories, *MI* myocardial infarction, *RRT* renal replacement therapy, *SSI* SYNTAX Score I

*Statistically significant at *P* ≤ 0.05

We also investigated such association, with the preoperative SSII, and found a statistically significant association of SSII with in-hospital mortality, overall 90 days mortality, and need for RRT, which summarized in Table 4.

**Table 4:** Relation between preoperative SSII with the mortalities and studied complications (*n* = 150)

	***N***	%	SSII CABG (mean ± SD)		***P***
			Complicated	Non complicated	
**In-hospital mortality**	8	5.3	36.92 ± 6.63	26.40 ± 10.78	0.007^*^
**90 days mortality**	10	6.7	33.64 ± 9.07	26.48 ± 10.84	0.043^*^
**MI**	10	6.7	28.68 ± 10.07	26.84 ± 10.93	0.606
**Stroke**	5	3.3	29.44 ± 7.82	26.87 ± 10.95	0.605
**Need for RRT**	3	2	42.40 ± 13.50	26.64 ± 10.61	0.012^*^
**Mediastinitis**	5	3.3	35.68 ± 4.60	26.66 ± 10.89	0.067

*P P* value for association between different categories, *MI* myocardial infarction, *RRT* renal replacement therapy, *SSII* SYNTAX Score II

*Statistically significant at *P* ≤ 0.05

We did univariable and multivariable analysis of the incidence of total morality and each of the studied complication, with the preoperative clinical data of our patients (age, sex, DM, LM disease, creatinine clearance, EF %, EuroSCORE II, SSI, and SSII) to study the association between them.

As regards the mortality, we found a statistically significant association of the overall mortality with SSI and SSII (*P* = 0.015, 0.048 respectively) in the univariable analysis; also, SSI appeared to be an independent risk factor for mortality (OR 1.192, 95% CI 1.018–1.396) (*P* = 0.029) in the results of multivariable analysis; these relations demonstrated in Table 5.

**Table 5:** Univariable and multivariable analysis for the parameters affecting overall mortality (for total sample)

Mortality	Univariable		^**#**^Multivariable	
	***P***	OR (95% CI)	***P***	OR (95% CI)
**Age**	0.391	1.034 (0.957–1.118)		
**Sex (female**^**(R)**^**, male)**	0.716	1.29 (0.32–5.23)		
**DM**	0.426	2.35 (0.286–19.31)		
**LM**	0.217	2.25 (0.62–8.19)		
**EF %**	0.691	1.016 (0.939–1.10)		
**Creatinine clearance**	0.223	0.980 (0.950–1.012)		
**EuroScore II**	0.546	0.738 (0.274–1.982)		
**SSI**	0.015^*^	1.209^*^ (1.038–1.409)	0.029^*^	1.192^*^(1.018–1.396)
**SSII**	0.048^*^	1.069^*^ (1.001–1.14)	0.114	1.059 (0.986–1.136)

*OR* Odd’s ratio, *CI* confidence interval, *SSI* SYNTAX Score I, *SSII* SYNTAX Score II

#All variables with *P* ≤ 0.2 was included in the multivariable

*Statistically significant at *P* ≤ 0.05

Table 6 shows that incidence of MI showed a significant associated with increased SSI (*P* = 0.020) in univariable analysis; also, SSI appeared to be an independent risk factor for MI, in the results of the multivariable analysis (OR 1.182, 95% CI 1.016–1.375).

**Table 6:** Univariable and multivariable analysis for the parameters affecting MI (for total sample)

MI	Univariable		^**#**^Multivariable	
	***P***	OR (95% CI)	p	OR (95% CI)
**Age**	0.191	0.959 (0.902–1.021)	0.712	0.985 (0.907–1.069)
**Sex (female**^**(R)**^**, male)**	0.716	1.29 (0.32–5.23)		
**DM**	0.998	–		
**LM**	0.888	0.905 (0.224–3.66)		
**EF**	0.158	0.945 (0.875–1.022)	0.514	0.972 (0.891–1.059)
**Creatinine clearance**	0.127	1.026 (0.993–1.060)	0.244	1.025 (0.983–1.069)
**EuroScore II**	0.955	1.021 (0.501–2.079)		
**SSI**	0.020^*^	1.195^*^ (1.029–1.387)	0.030^*^	1.182^*^(1.016–**1.375)**
**SSII**	0.603	1.016 (0.957–1.079)		

*OR* Odd’s ratio, *CI* confidence interval

#All variables with *P* < 0.2 was included in the multivariate

*Statistically significant at *P* ≤ 0.05

Incidence of mediastinitis showed a significant association with increasing age and EuroSCORE II (*P* = 0.019, 0.006 respectively), while need for RRT was significantly associated with EuroSCORE II, SSII, and preoperative creatinine clearance (*P* = 0.045, 0.006, 0.027 respectively) in their univariable analysis. In the multivariable analysis, none of the clinical data appeared to be an independent risk factor for mediastinitis or RRT. Stroke incidence, in our study, had no significant association with any of the clinical factors included in the analysis.

## Discussion

SYNTAX Score I is an anatomical scoring system that used for assessment of the complexity of coronary arteries and estimation of long-term mortality and MACCE post PCI; SSII combines both SSI and 8 clinical variables, for estimation of 4 years mortality following both CABG and PCI for individual patient and gives its recommendation about the best revascularization strategy for the patient (PCI, CABG, or both). The impact of these scores on the short-term results following CABG is still unclear.

We prospectively studied 150 patients with MVD, performed elective primary isolated CABG, to assess the impact of preoperative SYNTAX Scores on the short-term outcome. We followed up our patients for 90 days postoperatively (in hospital, visits, and phone calls), for onset of mortality, MI, stroke, mediastinitis, and need for RRT, then correlated the results obtained with the preoperatively calculated SYNTAX Scores and the preoperative clinical data. Table 2 summarizes the incidence of mortality and each of the studied complications in our study in 90-day follow-up period.

Does SSI, which reflects the anatomical complexity of the coronary arteries, affect the short-term outcome of CABG? This was the first question to be answered in our study. By reviewing the published studies in this topic, we found a little available data on the effect of SSI on the short-term results. Birim [10] and his colleagues searched the outcome of CABG in relation to SSI in 148 patients with LM disease, within 30 days and 1-year follow-up durations and found that the higher SSI was associated with greater incidence of mortality and MACCE within 30 days. Also, Alcazar [13] and his colleagues, when studied 716 patients with 3-vessel and/or LM disease, performed primary off pump CABG to evaluate the influence of SSI on both short- and long-term outcomes concluded that higher SSI is associated with higher in-hospital and long-term onset of MACCE but not with mortality.

In our study, we found a significant impact of SSI on short-term outcome, by founding a significant association between increased preoperative SSI and total 90 days mortality (mean ± SD = 30.75 ± 2.9) (*P* < 0.001), in-hospital mortality (mean ± SD = 31.63 ± 1.87) (*P* < 0.001), MI within 90-day follow-up (mean ± SD = 30.55 ± 4.79) (*P* = 0.015), and mediastinitis (mean ± SD = 27.70 ± 1.10) (*P* = 0.045).

Melina [14] and associates had another opinion, when they studied 191 patients with left ventricular dysfunction undergoing CABG with mean SSI was 32 ± 13, to assess the relation between the complexity of coronary arteries with short- and long-term outcome. They did not find a significant impact of SSI on the outcome in first 12-month follow-up but found that the greater complexity was associated with increased incidence of mortality and MACCE in 6-year follow-up duration. Moher [14] and his colleagues also found no impact of SSI on the postoperative outcome, within 2-year follow-up of SYNTAX cohort, who performed CABG in the original SYNTAX trial, and the outcome was related mainly to the completeness of revascularization, that was also, the main factor affected post CABG outcome in the study performed by Kato [15] and his working group, for the same purpose. These differences with our work may be due to the differences in study design, number of enrolled patients, associated comorbidities in each cohort, mean SSI for the study cohort, duration of the follow-up, and differences in the graft material and revascularization techniques.

The importance of SSII arises from its ability to individualize the decision making, by respecting the special patient-related clinical parameters that affect the outcome of surgery; Esper [11] and his colleagues performed a subgroup analysis of the patients enrolled in FREEDOM trial who received CABG and correlated their incidence of MACCE with preoperative SSI. They found a great impact of other factors, rather than SSI on the outcome of CABG in diabetic patients with MVD, as age and associated comorbidities. Finally, they recommended that SSI should not be used as the sole guiding tool, in choosing the revascularization strategy in diabetic patients and MVD.

In a multicenter trial conducted on 2961 patient performed isolated primary CABG, Gonzales [9] and his colleagues tried to assess the performance of STS Score, EuroSCORE II, and SSII in the prediction of both 30 days and 4 years mortalities. They found a better performance of EuroSCORE II and STS Score in prediction of 30 days mortality, while SSII was the most powerful predictor of long-term mortality.

Misumida [16] and his associates tested the prognostic value of SSII, when they retrospectively reviewed the data of 286 American Veterans with 3-vessel and/or LM disease performed either CABG or PCI. They compared the predicted 4 years mortality from SSII calculations with the observed 4 years mortality for the study population and found a well performance of SSII in estimation of 4 years mortality following both CABG and PCI at low and intermediate scores and underestimation of mortality in PCI group only with high scores.

In our study, we also found a prognostic value of preoperative SSII in evaluating the post CABG short-term outcome, as we found a significant association between increased preoperative SSII and in-hospital mortality (mean ± SD = 36.92 ± 6.63) (*P* = 0.007), total 90 days mortality (mean ± SD = 33.64 ± 9.07) (*P* = 0.043), and need for RRT (mean ± SD = 42.40 ± 13.50) (*P* = 0.012).

Should we respect SSII recommendations? Modolo [17] and his colleagues answered this question, when they reviewed SSII recommendations for the patients involved in EXCEL trial, and compared it with the actual treatment received, expected, and observed all causes mortality within 4 years. They found a higher observed mortality in the patients randomly assigned to PCI with SSII CABG recommendation, in comparison with their expected post CABG mortality, and reach the conclusion that non respect SSII CABG treatment recommendation was associated with higher mortalities. In our study, all patients were assigned to CABG with 14 of them had SSII PCI recommendation; among these patients, we had one in-hospital mortality from perioperative MI (7.1%).

Analysis of mortality in our study showed that we had 5.3% in-hospital mortality and 6.7% total 90 days mortality. Our incidence was near to the results of Alberto [18] and his colleges, when they investigated the risk factors of mortality in their series of 1628 CABG patients within 30-day follow-up. They had 30 days mortality rate 8.7%, with dialysis and neurological dysfunction were the main associated risk factors, while in our study MI was the main associated complication.

Also, our total mortality showed a significant association with SSI and SSII (*P* values were 0.014 and 0.048 respectively) in the results of its univariable analysis with the preoperative clinical data, while SSI appeared to be independent risk factor for mortality in the multivariable analysis (OR 1.192, 95% CI 1.018–1.396).

Diagnosis of MI in the perioperative period is challenging, with a wide range of incidence in the literatures, according to the methods and definitions used in its diagnosis. This point in particular, approved by Belly-Cote [19] and his colleges, when they used different biomarkers (CKMB, Troponins), to study the differences in both incidence and prognosis of post CABG MI, when different definitions based on different biomarkers used in its diagnosis; they found that not only the incidence was different, but also, the prognosis according to the biomarker that used for definition of MI.

Diagnosis of perioperative MI does not depend on a single parameter; a combination of ECG changes (ST elevation, new Q wave, new LBBB) with hemodynamic instability, ventricular arrhythmias, elevated biomarkers, and/or new RWMA in the echo, is diagnostic. In these cases, early coronary angiography is very important to confirm the diagnosis, detect the etiology (graft or non-graft related causes), and provide treatment (ad hoc PCI) [19–21].

In our study, we used CKMB/total CK ratio > 10%, as the biomarker for diagnosis of perioperative MI; this biomarker used also by Rupperchet [21] and his working group, when they retrospectively studied 108 patients, suffered from post CABG ischemia and performed early postoperative coronary angiography, to evaluate the impact of early coronary angiography in improving the outcome in the cases associated with post CABG ischemia.

We had a total incidence of MI in 90-day follow-up 6.7% and 4.6% in the perioperative period. In PREVENT IV study which included a total number of 3014 patients performed CABG, the investigators studied the incidence, mechanisms, and prognosis of MI in both perioperative period and 2-year follow-up; they found the incidence of perioperative MI in their series 9.8% [22].

All the previously mentioned studies and the recent guidelines on myocardial revascularization emphasize on the importance of coronary angiography, in cases with post CABG ischemia to improve the outcome. In our study, we relied only on the clinical, biochemical, and echo changes in diagnosis of MI in the perioperative period (lacking the facility of intraoperative or early postoperative angiography in our institute) and coronary angiography done for the patients who presented with late onset MI after hospital discharge. This issue demonstrates high incidence of mortalities, among the patients with MI in our series [3, 19–22].

Univariable analysis of MI incidence in relation to the preoperative clinical data showed a significant association with SSI (mean ± SD = 30.55 ± 4.79) (*P* = 0.020) that also appeared to be an independent risk factor for MI in the results of multivariable analysis (OR 1.182, 95% CI 1.016–1.375) (*P* = 0.030).

We did not find recent papers that discuss the association between SSI and perioperative MI as a separate entity, but many authors included MI as one of the components of MACCE in their studies of the relation between SSI and post CABG outcome as Birim [10], Alcazar [13], Melina [23], Mohr [14], and their colleagues. Their results already compared with our results in a previous part of discussion.

Lack of studies that investigate the relation between perioperative MI as a separate entity and SSI is considered as a point of strength in our study.

We had 3.3% of our patients stroked in 90-day follow-up period and 2.6% in the perioperative period; these numbers are near to the incidence in the literatures. We did not find a significant association between stroke incidence in our study population and any of the preoperative clinical factors included in the analysis [24].

The need for RRT in our study occurred in 2% of cases (3 patients), with no mortality among them. Ivert [25] and colleagues, on their series of 28,220 patients with primary isolated CABG, 0.6% of their patients needed a postoperative dialysis with 30 days mortality rate, among them was 26%. Ranucci [26] and his working group, during their analysis of 7675 adult patient undergoing on pump cardiac surgery, showed that 1.7% of their patients needed dialysis with mortality rate, among them was 46.9%. Absence of RRT associated mortality among our patients may be explained by the low number of cases, with a little chance for mortality to appear and/or early initiation of RRT in these patients that proved to be a very important factor in the reduction of mortality and improving the outcome, which is approved by a recently published meta-analysis on this topic [27].

Preoperative creatinine clearance, EuroSCORE II, and SSII of our study population showed a significant association with need for RRT (*P* values = 0.045, 0.006, 0.027 respectively) when the univariable analysis of RRT with the preoperative clinical data was done, but none of them appeared as independent risk factor for RRT in the result of multivariable analysis.

Incidence of mediastinitis among our patients was 3.3%; this incidence coincides with the results of Oliviera group [28] when they studied 1322 cases undergoing CABG and found the incidence of mediastinitis among their patients was 4.2%.

The results of univariable analysis of mediastinitis incidence with the preoperative clinical data showed a significant association between mediastinitis with age and EuroSCORE II (*P* = 0.019, 0.006 respectively), but none of them could be considered as independent risk factor of mediastinitis.

The strong relation between SSI and short-term outcome especially mortality and MI in our study may be explained by increased anatomical complexity of CAD is associated with technical difficulties as increased procedure time, number of grafts, and need for endarterectomy with target vessel reconstruction; all these are associated with increased mortality and morbidity. Also, increased SSI is always associated with poor distal run off in the native coronaries that contributes to a subsequent graft occlusion. These factors explain the higher incidence of mortalities and MI associated with high SSI (short-term complications that related mainly to anatomical complexity and technical factors).

Despite increased SSII is associated with significant increase in the mortality, it is less powerful than SSI in prediction of short-term post CABG outcome. We explained it by the fact that SSII value is affected by both SSI and the clinical situation of the patient. For example, a patient with high SSI and few comorbidities will have low SSII, while those with low SSI and multiple comorbidities will have high SSII. On the other hand, increased SSI is associated with more technical difficulties and procedure time with their impact on short-term outcome and vice versa.

## Conclusion

Both SYNTAX Scores are good predictors of short-term outcome after CABG; increased preoperative SSI (anatomical complexity of coronary lesions) in the patients undergoing CABG, was associated with increased postoperative mortalities (in-hospital and total 90 days), MI, and mediastinitis, in our study population. SSI also appeared to be an independent risk factor for short-term mortality and MI following CABG.

Increased preoperative SSII was associated with increased postoperative mortalities (in-hospital and total 90 days) and need for RRT in the patients undergoing CABG. We should also respect and follow the recommendations of SSII calculator, in deciding the revascularization strategy.

## Data Availability

The datasets used and/or analyzed during this study are available from the corresponding author on reasonable request.

## Abbreviations

CABG: Coronary artery bypass grafting surgery
CAD: Coronary artery disease
CK: Creatinine kinase
CKMB: Creatinine kinase isoenzyme MB
COPD: Chronic obstructive pulmonary disease
ECG: Electrocardiogram
EF: Ejection fraction
EuroSCORE II: European System for Cardiac Operative Risk Evaluation II
LM: Left main
MACCE: Major adverse cardiac and cerebrovascular events
MI: Myocardial infarction
MVD: Multi-vessels disease
PCI: Percutaneous coronary intervention
PVD: Peripheral vascular disease
RRT: Renal replacement therapy
RWMA: Regional wall motion abnormalities
SSI: SYNTAX Score I
SSII: SYNTAX Score II
STS Score: Society of Thoracic Surgeons risk score
SYNTAX: Synergy between PCI with Taxus and Cardiac Surgery
ANOVA test: Analysis of variance test
